# Bioconversion of Sugarcane Vinasse into High-Added Value Products and Energy

**DOI:** 10.1155/2017/8986165

**Published:** 2017-11-08

**Authors:** Bianca Ferrazzo Naspolini, Antonio Carlos de Oliveira Machado, Walter Barreiro Cravo Junior, Denise Maria Guimarães Freire, Magali Christe Cammarota

**Affiliations:** ^1^National Institute of Technology (INT-MCTIC), Av. Venezuela, No. 82, Centro, 22453-900 Rio de Janeiro, RJ, Brazil; ^2^National Service for Industrial Apprenticeship, Technology Center of Chemical and Textile Industry (SENAI CETIQT), Rua Magalhães Castro, No. 174, Riachuelo, Rio de Janeiro, RJ, Brazil; ^3^Institute of Chemistry, Federal University of Rio de Janeiro, Av. Athos da Silveira Ramos, No. 149, Bloco A, Sala 549-1, Cidade Universitária, 21941-909 Rio de Janeiro, RJ, Brazil; ^4^School of Chemistry, Federal University of Rio de Janeiro, Av. Athos da Silveira Ramos, No. 149, Bloco E, Sala 115, Cidade Universitária, 21941-909 Rio de Janeiro, RJ, Brazil

## Abstract

Vinasse, a residue from bioethanol production containing high organic matter concentration, was used as substrate in submerged fermentation of* Pseudomonas aeruginosa* PA1 for biosurfactant production. About 2.7 g/L of rhamnolipids was obtained, with surface tension of 29.2 mN/m and critical micelle concentration of 80.3 mg/L. After separation of rhamnolipid and biomass, residual fermentation media were submitted to anaerobic biodegradation in mesophilic conditions. The residual medium derived from fermentation with vinasse diluted to 1 : 1, without addition of nitrogen, C : N 21, and for 168 h, led to 63.2% chemical oxygen demand (COD) removal and 97.6 mL CH_4_/g COD_removed_. Compared to results obtained with fresh vinasse (73.7% COD removal and 112.4 mL CH_4_/g COD_removed_), it could be concluded that both processes can be integrated in order to add value to the residue and obtain energy, reducing production costs and at the same time environmental impacts related to vinasse disposal.

## 1. Introduction

The increase of bioethanol production has led to increased vinasse generation, which is a byproduct of the distillation step subsequent to fermentation of carbohydrates obtained from different sources of saccharides materials (sugarcane and beet), starchy materials (maize, wheat, rice, cassava, and oat), and lignocellulosic materials (sugarcane bagasse, straw, and wood, among others) [1]. In Brazilian plants, for every liter of ethanol produced, on average 13.7 liters of vinasse are generated [2]. Considering a total production of 30 billion liters of ethanol in the 2015/16 crop [3], the average vinasse production was approximately 411 billion liters.

Vinasse has variable chemical composition, which depends mainly on the raw material used in the bioethanol production [4], and its main features are dark brown color, acidic pH (3.5–5.0), and high temperature (80–100°C), organic matter concentration (COD 50–150 g/L), and salinity (K, Ca, and Mg) [5, 6].

These characteristics, combined with high production volume, necessarily require adequate treatment and final disposal. In Brazil, the most common vinasse disposal is its application to the soil of sugarcane fields as fertilizer due to its high levels of organic matter and nutrients (especially potassium but also nitrogen and phosphorus). According to economic perspectives, this alternative is the simplest and cheapest solution for the disposal of such abundant effluent [7, 8], as it demands a small initial investment and low maintenance costs, has no technological complexity, is rapid application, and increases crop yields. However, adverse environmental impacts, such as salinization and leaching of soil nitrates [9], contamination of surface waters [10], and even the worsening of global warming by releasing nitrous oxide (N_2_O) in the soil heterotrophic denitrification [11], should be considered.

The use of vinasse as raw material for biosurfactant production (rhamnolipids) in the stage previous to its treatment and disposal seems to be an interesting alternative technology, since it helps reduce the organic matter concentration introduced into the soil and, at the same time, obtains a high-value product with vast potential in industrial applications [12, 13]. In general, agroindustrial waste containing high levels of carbohydrates can serve as a carbon source for biosurfactant production [14]. The use of these alternative substrates presents some challenges, such as the difficulty of finding the right nutrient composition that enables the synthesis of the product of interest, residue standardization (due to natural composition variations), and costs inherent to the process. Such use, however, can reduce the production costs of biotechnological routes at competitive levels compared to similar biosurfactants obtained by petrochemical routes, while reducing the environmental impacts related to waste disposal [15].

After biosurfactant extraction, the residual fermentation medium still has high concentrations of carbon and nutrients, which must be reduced before being discharged into the environment. Faced with currently available treatment technologies, anaerobic digestion seems to be interesting because it presents environmental and energy advantages such as low operation costs, reduced sludge production, and methane formation, which has high combustion heat [16, 17]. After anaerobic digestion, vinasse may have less organic load but still contains nutrients and minerals that can be used as fertilizer [18].

In order to improve the energy potential and bioethanol production sustainability, wastewaters generated from ethanol production should not be considered as process waste. Therefore, the use of these wastes should be consolidated so that they become raw materials for other processes. This concept is now inherent to the field of biological treatment of industrial effluents, in which scientific and technological advances made in recent years have driven the creation of new research lines aimed at not only the adequacy of waste disposed into the environment but also the recovery of energy and products from these effluents. Through this approach, the wastewater is considered as a raw material for the biotechnological process that can generate energy and value-added products, playing a primary role in controlling environmental pollution [17, 19]. A promising technology that has been studied to harness the energy produced by microorganisms from anaerobic oxidation of biodegradable organic compounds is microbial fuel cells (MFCs). Several of these systems are studied for the treatment of wastewater and for the recovery of value-added products such as bioflocculants, bioplastics, biosurfactants, hydrogen, methane, and even electricity [20].

Energy recovery and production of various products are principles inherent to the biorefinery concept, which has been recently developed. Biorefinery integrates the process of biological production of fuels, bioenergy, and biomass byproducts, analogous to an oil refinery. This technological possibility is now reconsidered due to the need to reduce the organic matter concentration of vinasse while nutrients and mineral content remain for use/disposal in soil, as well as the interest in optimizing the energy balance of sugarcane biorefineries [18, 21].

According to Moraes et al. [22], biogas could replace up to 40% of the annual diesel supply in the agricultural operations of a sugarcane biorefinery and still provide approximately 14 MWh annually from cogeneration. Yeast drying would be another economically attractive use for biogas in a biorefinery. About 55% of the internal return rate would be achieved with the sale of dried yeast, which is grown as animal supplementation [18].

The aim of this study was to evaluate the use of vinasse as a culture medium for biosurfactant production (rhamnolipid) via submerged fermentation of* Pseudomonas aeruginosa* PA1 and residual fermentation medium for methane production.

## 2. Materials and Methods

### 2.1. Vinasse Origin and Characterization

Vinasse used in this study came from a first-generation ethanol plant (1G ethanol from fermentation of sugarcane juice) located in São Paulo (Brazil), collected in sufficient amount for all tests performed, and stored in 50 L gallon at room temperature. Chemical oxygen demand (COD), pH, biochemical oxygen demand (BOD), total suspended solids (TSS), and chloride were determined according to methods described in Standard Methods [23]. Total nitrogen and total organic carbon (TOC) were quantified in Shimadzu TOC TNM-1 analyzer. Total phosphorus and sulfate were determined by colorimetric methods using methodology and HACH® kits. Alkalinity and volatile acids were quantified by potentiometric methods, as described by Dilallo and Albertson [24] and Ripley et al. [25], respectively.

### 2.2. Vinasse Valorization as Substrate for Biosurfactant Production

After determination of C, N, and P concentrations, vinasse was used as raw material for biosurfactant production (rhamnolipid) through submerged fermentation of* Pseudomonas aeruginosa* PA1 in conventional medium and medium with vinasse. The medium with vinasse employed raw vinasse diluted with distilled water and pH adjusted to 7.0 due to its high salt concentration and very acidic pH.

*Pseudomonas aeruginosa* PA1, previously isolated from oil wells in Northeast Brazil, was preserved in an ultrafreezer in 10% glycerol solution. For the strain storage, the bacterium was initially grown in medium with composition (in g/L): NaNO_3_ 1, KH_2_PO_4_ 3, K_2_HPO_4_ 7, MgSO_4_·7H_2_O 0.2, yeast extract 5, peptone 5, and glycerol 30. Growth was conducted in shaker at 30°C and 200 rpm for 40 h (in the exponential growth phase). Then, cells were stored in cryogenic vials at −80°C in 15% glycerol (v/v).

To prepare inocula, strains stored at −80°C (1 cryotube per flask) were directly inoculated into 1000 mL Erlenmeyer flasks containing 300 mL of culture medium (cited above). About 40 h after activation of cells at 30°C and 200 rpm, the material was centrifuged (5000*g* for 10 min) and resuspended to inoculate the culture medium.

Biosurfactant production in conventional culture medium was conducted in 1000 mL Erlenmeyer flasks with 300 mL culture medium with the following composition (g/L): glycerol 30, NaNO_3_ 1.2, K_2_HPO_4_ 7, KH_2_PO_4_ 3, and MgSO_4_·7H_2_O 0.2 [26]. The C : N ratio of the medium is about 60 mol/mol, but it can be modified by adjusting glycerol and NaNO_3_ concentrations. Media were inoculated with an initial standard cell concentration of 0.5 g/L, and fermentation was conducted at 30°C/200 rpm, with cell growth, glycerol consumption, and rhamnolipid production monitoring. Fermentation terminated when the carbon source was depleted.

After fermentation, the pH of the fermentation medium was lowered to 2.0 by adding HCl 1 mol/L for rhamnolipid precipitation. The medium was then centrifuged at 4°C/10,000 rpm for 10 min to separate solid and liquid phases. In the solid phase, 50 mL of distilled water was added to solubilize the precipitated material, and the pH was adjusted to 7.0 with NaOH 1 mol/L. The liquid phase had pH adjusted to 7.0 with NaOH 1 mol/L. The biosurfactant obtained in fermentation conducted in conventional medium was called BS-C.

Eight fermentation media were evaluated using vinasse as feedstock supplemented with glycerol as carbon source and NaNO_3_ or NH_4_OH as nitrogen source at different fermentation times. Glycerol and nitrogenous salts were added to adjust the initial C : N ratio to 15, 21, and 60 mol/mol in medium consisting of vinasse, which was diluted with distilled water to 1 : 1 and had pH adjusted to 7.0 with NaOH due to its high salt concentration and very acidic pH (Table 1). In some fermentations due to the high concentration of suspended solids in the sample, vinasse was centrifuged before being diluted.

Fermentation conditions were the same as those described for conventional medium fermentation. The rhamnolipid was separated from the fermented medium by acid precipitation and centrifugation, and the residual fermentation medium was characterized and used in anaerobic digestion assays. The biosurfactant obtained by vinasse fermentation was called BS-V. After separation of the biosurfactant produced, only six residual media (media 2, 3, 4, 6, 7, and 8) were transferred to anaerobic digestion.

### 2.3. Evaluation of the Residual Fermentation Medium for Methane Production

Anaerobic biodegradation tests were conducted on 100 mL penicillin flasks with working volume of 90 mL consisting of anaerobic sludge and raw vinasse (as collected) or residual fermentation medium from biosurfactant production. Both media were diluted to an initial COD of about 4000 mg/L. The sludge used as inoculum was obtained from a mesophilic UASB (upflow anaerobic sludge blanket) reactor in operation in a poultry slaughtering industry and the samples collected had concentrations of volatile suspended solids (VSS) of 22–32 g/L. The sludge volume added to each flask was calculated to maintain a COD (initial vinasse) : VSS (sludge) ratio of 1 : 1.

When necessary, raw vinasse and residual fermentation medium were supplemented with NH_4_Cl and KH_2_PO_4_ to obtain COD : N : P ratio of 350 : 5 : 1 [27]. The pH of raw vinasse and residual media was adjusted to 7 by adding NaHCO_3_. Aliquots were collected to determine the initial COD prior to mixing with the anaerobic sludge. Flasks were sealed with rubber stoppers and aluminum seals and incubated in room at 30°C up to stabilization of the accumulated biogas volume, which was measured by displacement of the piston of 60 mL graduated plastic syringes connected to the flasks.

Each condition was performed in five replicates, and the results are presented as mean ± standard deviation. Biodegradability was evaluated by COD removal efficiency and biogas production. Final COD was determined on the last day of biodegradability test when biogas production was stabilized. Biogas was collected in gasometric ampules and directly injected into Varian Micro GC 4900 chromatograph for methane determination. The conditions used in chromatography were as follows: PPQ column of 10 m × 0.32 mm, column temperature of 50°C, thermal conductivity detector, detector temperature of 250°C, injector temperature of 80°C, helium as carrier gas, and analysis time of 1.5 minutes.

## 3. Results and Discussion

### 3.1. Vinasse Characterization

The characteristics of the raw vinasse used in the composition of the biosurfactant production medium are presented in Table 2. Wilkie et al. [1] analyzed the characteristics of vinasse obtained from different raw materials, emphasizing that such wastes exhibit wide variations in COD (85 ± 31 g/L) and BOD values (39 ± 11 g/L) and nutrients, with 1230 ± 630 mg/L of nitrogen and 190 ± 35 mg/L of phosphorus. Sulfate concentrations are also very high (3500 ± 2500 mg/L), and pH values are in the range of 4-5. The vinasse used in this study, and considering the standard deviations, showed little difference, except for the BOD and phosphorous concentration, which are greater and lower in this study, respectively.

The pH (4.1) of the vinasse is very low and unfavorable for biosurfactant production, thus requiring adjustment to more appropriate values. Santos et al. [28] evaluated the effect of the culture medium pH on rhamnolipid production using* P. aeruginosa* PA1 and found that the best value was 7.0. Using culture medium based on glycerol and nitrate, Jamal et al. [29] obtained highest rhamnolipid production by another* P. aeruginosa *at pH 7.3.

Vinasse has high organic matter concentration (COD of 77 g/L), predominantly soluble (78% of total COD is in the soluble form), and biodegradable (BOD of 57.6 g/L, 75% of COD). The COD/BOD ratio of 1.34 indicates high biodegradability and that the vinasse constituents may be used by bacteria as substrate for biosurfactant production. Kaskatepe and Yildiz [14] reported some studies on rhamnolipid production using agroindustry residues as substrates, mostly conducted with molasses, a sugar production industry byproduct with high sucrose concentration (50–55% w/w). The use of vinasse as a substrate was only found in the study by Oliveira and Garcia-Cruz [30], which obtained 27.7 g/L of biosurfactant in the fermentation of* Bacillus pumilus *with 5% vinasse after 48 h.

The soluble COD concentration, compared to total COD, shows that about 17 g/L of organic matter is in the particulate form, consistent with the TSS concentration which is probably of organic origin. The presence of organic particulates may not favor the biosurfactant production, as they may present lower biodegradation rates and interfere with the enzymatic activity. Oliveira and Garcia-Cruz [30] removed insoluble solids from vinasse by filtration before preparation of the fermentation medium. In this study, the use of vinasse after removal of suspended solids resulted in higher rhamnolipid production.

Vinasse has high concentration of volatile acids (13,080 mg/L as acetic acid) and no alkalinity, which is unfavorable for rhamnolipid production because it results in low pH values, as previously mentioned. This condition is also unfavorable for the anaerobic biological process, because the interaction of alkalinity with volatile acids is based on the ability of the system to neutralize acids formed in the process and to buffer pH in a possible accumulation of volatile acids [27].

Vinasse also contains macronutrients required for microorganisms in both biosurfactant production and anaerobic biological processes. However, although the C/N ratio has a value (18.5 mol/mol) close to that recommended in other studies such as C/N ratio of 18 mol/mol [29, 31], the need for a carbon source to induce biosurfactant production, such as glycerol [32], involves the adjustment of initial carbon and nitrogen concentrations. The addition of glycerol would be a way to take advantage of an abundant source of waste, in this case a biodiesel industry coproduct.

The high salinity of raw vinasse, represented by concentrations of sulfates and chlorides, can interfere with biosurfactant production. In this study, vinasse was diluted to reduce such effects. However, a previous adaptation of* P. aeruginosa* can lead to greater resistance to higher salt concentrations. In literature, there are reports of biosurfactant production under extreme pH, temperature, and salinity conditions. Elazzazy et al. [33] isolated a* Virgibacillus salarius* strain, in which largest lipopeptide-type biosurfactant production (1.6 g/L) occurred in the presence of 4% (w/v) NaCl.

Given the need to treat and properly dispose of this industrial wastewater, the use of vinasse as raw material for rhamnolipid production is a technological alternative. After all, the effluent has high concentrations of carbon (TOC, COD, and BOD), nutrients (N, P), and salts, which can be recovered before final disposal into the environment.

### 3.2. Biosurfactant Production in Conventional Medium and Medium with Vinasse

**Figure 1:**
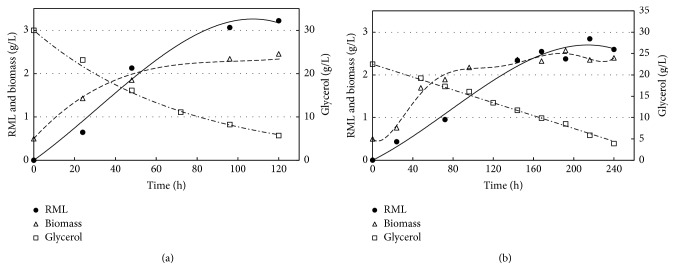
Glycerol consumption kinetics, microbial growth, and rhamnolipid production in fermentations with C : N = 15 mol/mol at 30°C/250 rpm. (a) Conventional medium and (b) medium containing vinasse diluted to 1 : 1.

Figure 1 shows the kinetics of substrate consumption, microbial growth, and rhamnolipid production observed in one of the fermentations conducted with both culture media. Fermentations shown in this figure were conducted under the same cultivation conditions and with the same initial C : N ratio (15 mol/mol), in which the medium with vinasse required adjustment by adding glycerol and NaNO_3_ (medium 8, Table 1).  Table 3 shows parameters of fermentation processes and characteristics of biosurfactants produced in both ways.

Although biomass and rhamnolipid production showed similar values: 1.9 and 2.7 g/L, respectively, in medium with vinasse, and 2.0 and 3.2 g/L in conventional medium, the yield was lower in medium with vinasse. The chemically defined medium yielded 0.027 g/L·h, while, in medium containing vinasse, this parameter was 0.011 g/L·h or 59% lower. This lower yield is probably due to the slower glycerol consumption in medium with vinasse, according to the glycerol consumption rates shown in Table 3. In the chemically defined medium, glycerol is consumed at a rate 2.5 times greater than in medium with vinasse. Probably the slower glycerol consumption in medium with vinasse is due to the assimilation of carbon sources present in the vinasse by bacteria.

The characteristics of biosurfactant produced by submerged fermentation with* Pseudomonas aeruginosa* PA1 in conventional cultivation (BS-C) and in dilute raw vinasse (BS-V), shown in Table 3, have similar values. Therefore, the application of BS-V must provide results similar to those obtained with BS-C. The values obtained in this study for surface tension (29 mN/m) and CMC (80 mg/L) in supernatants of fermentation media indicated that* P. aeruginosa* PA1 is able to use vinasse (50% v/v) as a source of carbon and nutrients. The values obtained are much lower compared with those obtained by Oliveira and Garcia-Cruz [30], who produced biosurfactant with* Bacillus pumilus* with surface tension of 45 mN/m and CMC of 1500 mg/L in medium containing only 5% (v/v) vinasse.

In fermentations conducted with* P. aeruginosa* PA1, Santa Anna et al. [32] obtained 1.7 g/L of rhamnolipid using initial cell concentration of 0.004 g/L and glycerol as substrate at C : N ratio of 22.8. Santos et al. [28] optimized the production process with the same strain and culture medium of this study and obtained 10.9 g/L of rhamnolipid in batch fed with C and N limitation. After optimization of the rhamnolipid production by another* P. aeruginosa* strain, Jamal et al. [29] obtained 4.44 g/L after 72 h of fermentation using C : N ratio of 17.5 and 2.8% inoculum.

Values obtained (Table 3) and compared with literature [28] indicated a need for studies to enable better adaptation of the strain to the vinasse components (reduction of lag phase) and increased rhamnolipid productivity. However, the use of vinasse as a fermentation medium for rhamnolipid production proved to be feasible in comparison with conventional cultivation medium.

A comparison of consumption and cost of chemicals in conventional fermentation medium (BS-C) and fermentation medium based on vinasse (BS-V) is shown on Table 4. This comparison was performed for 1-ton rhamnolipid produced, considering the consumption of glycerol and rhamnolipid production shown in Table 3. The unit value of each item considered in the costs was obtained from suppliers of commercial chemicals. In both fermentations, the highest cost is the source of carbon glycerol, used in this cost estimate as a high purity commercial product. However, replacing part of the glycerol and nutrients (such as P, Mg, and others) in the conventional fermentation medium by the vinasse constituents provides a marked reduction in production costs. This even considers the cost of the alkalis required to adjust the pH of the raw vinasse (average 4.1) to the initial pH of the fermentation (pH 7.0), which is not necessary in the conventional fermentation. In addition to reducing costs related to the consumption of chemicals, the use of vinasse would reduce the consumption of clean water. If the glycerol from the biodiesel industry is used, the fermentation with vinasse could have even lower costs.

### 3.3. Evaluation of Residual Fermentation Media for Methane Production

Table 2 shows the characterization of residual fermentation media after separation of cells and biosurfactant. The values obtained varied according to the conditions adopted in fermentations. An analysis of their composition aiming at anaerobic digestion was performed. The pH of the fermented media after rhamnolipid precipitation had to be adjusted with NaHCO_3_ to values between 6.4 and 6.9, which are more suitable for anaerobic digestion. Hydrolytic bacteria that act in the early anaerobic digestion stages occur in a wider pH range, while methanogenic archaea responsible for methanogenesis survive only in the pH range of 6.6–7.4. Values outside this range result in low methane production, requiring the addition of alkalizing agents to maintain pH within the desired range [34].

The consumption of NaOH to adjust the pH of raw vinasse (between 4 and 5) to the initial fermentation value (about 7) and HCl for biosurfactant precipitation at the end of fermentation and again of NaHCO_3_ to adjust pH to values suitable for anaerobic digestion is a disadvantage of the integrated process proposed in this study, not only in terms of product costs (acids and alkalis) but also in terms of increased salinity (chloride and sodium) of vinasse after fermentation, which could affect the anaerobic process. However, raw vinasse also requires pH adjustment prior to anaerobic treatment, consuming alkali. Other procedures to recover biosurfactant produced without addition of chemicals must be studied to reduce costs and salinity of the fermentation medium.

The recovery of biosurfactants from fermentation media involves solvent extraction, ammonium sulfate or acid precipitation, crystallization, and centrifugation. Rhamnolipids, for example, are recovered by acid precipitation or extraction with ethyl acetate. These conventional methods present drawbacks, such as toxicity and high cost, which prevents industrial production. Thus, research has been directed towards the development of low cost extraction and purification procedures, to avoid the use of dangerous and expensive organic solvents [35]. Desai and Banat [36] reported different biosurfactant recovery methods for greater recovery and purity without addition of chemicals in the fermentation medium such as foam removal and collection outside the bioreactor, adsorption by resins, and membrane filtration.

TSS concentrations in fermented media ranged from 334 to 639 mg/L, with mean and standard deviation of 466 ± 114 mg/L. Despite the high variability, values are considered low and should not harm the anaerobic biological treatment. van Haandel and van der Lubbe [37] indicate tolerances for affluent TSS in high-rate anaerobic reactors of up to 500 mg/L or 10% of particulate COD (EGSB reactors, expanded granular sludge blanket) and up to 20% of particulate COD (UASB reactors, upflow anaerobic sludge blanket). Part of the reduction in VSS concentration compared to the initial concentration in the raw vinasse diluted to 1 : 1 (3297 mg/L) is due to the centrifugation process used to separate the produced rhamnolipid, a step that on an industrial scale may increase the process cost, which is another reason to search for innovative methods of biosurfactant recovery.

Through the BOD_5_/COD ratio, it could be inferred that the vinasse biodegradability following fermentation for biosurfactant production is reduced. Vinasse shows a ratio of 0.5–0.6, while, for raw vinasse, this ratio was 0.75. However, the values obtained in fermentation media still indicate a high degree of biodegradability. Comparing the BOD_5_ value of raw vinasse diluted to 1 : 1 (28,800 mg/L) with the average value obtained after fermentation (9,180 mg/L), there was a reduction of 68% due to the consumption of readily biodegradable compounds contained in the vinasse by* P. aeruginosa* bacteria during fermentation.

Residual media showed similar total and soluble COD values, with averages of 30,324 mg/L and 24,747 mg/L, respectively. Compared to values obtained for the raw vinasse diluted to 1 : 1, 38,484 mg/L and 30,198 mg/L, these values denote reduction of total and soluble COD of 21% and 18%, respectively. Such reductions would imply less organic matter to be converted into methane; however, vinasse volumes in Brazil are very high and could generate a substantial amount of energy. According to Moraes et al. [22], a single plant can process 2 million tons of sugarcane per season and could generate 18 MW per season from biogas, considering 60% (v/v) methane in the biogas and low heating value of 21,500 kJ/Nm^3^. Considering all vinasse produced in Brazil, this potential would reach 3,500 MW per season.

Another important point is that the value of the produced biosurfactant can be equal to or greater than the energy lost by the reduction of organic matter consumed in its production. The market price of rhamnolipid (R-95, 95%) produced by Agae Technologies (USA) is US$ 227/10 mg, and it is expected that the global market for biosurfactants will reach US$ 2.3 billion by 2020 [38]. Randhawa and Rahman [39] made a critical analysis of the market for biosurfactants and reported that if rhamnolipids become economically sustainable, nothing can prevent these biomolecules from dominating the market for surfactants. To this end, studies should be directed to high-producing strains, bioreactor technology, and cheaper substrates.

Henkel et al. [15] reported that the rhamnolipid production process will be economically viable in the near future, especially if renewable raw materials are adopted. The authors estimate the rhamnolipid production cost using various substrates obtaining sucrose from sugarcane at a cost of € 0.87/kg rhamnolipid, while, with molasses (containing 60% sucrose), the cost would be € 0.55/kg rhamnolipid. With raw glycerin from the biodiesel industry (containing 80% glycerol), the cost would be only € 0.21/kg rhamnolipid. The average rhamnolipid production in batch cultivations yields 0.1–0.62 g rhamnolipid/g substrate. Therefore, depending on substrate and process, more (up to 10 times) substrate is consumed in the production process than the amount of rhamnolipids synthesized. Therefore, in addition to the need to improve efficiency with the development of strategies to control the fermentation process, the use of low cost substrates (raw materials or waste) dramatically affects the production costs of biosurfactants [15].

The total phosphorus concentration in the residual media was very low (5–30 mg/L), with various media having values below the detection limit of the analytical method. The total nitrogen concentrations were higher and varied greatly due to the addition of different salts and concentrations of nitrogen sources to correct the C : N ratio in the fermentation media. In most fermentation media, the values obtained were lower than those for raw vinasse diluted to 1 : 1, indicating that nitrogen and phosphorus contained in the vinasse were consumed in the fermentation process. For adequate anaerobic digestion, COD : N : P ratio of 350 : 5 : 1 is recommended [27]. Thus, as vinasse following fermentation (average COD of 30,324 mg/L) requires 433 mg N/l and 87 mg P/l, only phosphorus supplementation would be necessary.

The sulfate concentration in the residual media ranged from 1600 to 11,600 mg/L. However, one must consider the COD/sulfate ratio, which when it is less than 7 g/g, it indicates strong inhibition of the methanogenic activity by sulfide produced by sulfate-reducing bacteria, and above 10 g/g indicates that most of H_2_S is removed from the liquid phase due to an intense biogas production, decreasing its inhibitory effect in the liquid phase [27]. Therefore, the COD/sulfate ratio in the residual media between 4.0 and 16.6 g/g indicates that the sulfide production may occur, damaging the anaerobic process. Sulfate present in residual media can lead to the formation of 533–3867 mg/L of sulfide. Inhibitory levels in literature range from 100 to 800 mg/L as dissolved sulfides or 50–400 mg/L as undissociated H_2_S, which can diffuse into the cell, denaturing proteins, interfering with the assimilatory sulfur metabolism, and reducing COD removal and methane yield [40]. However, inhibition by high sulfate concentrations is a problem widely known in anaerobic digestion of raw vinasse. España-Gamboa et al. [41], for example, attributed the low methane yield obtained, 0.263 m^3^  CH_4_/kg COD_added_ (compared to the theoretical yield of 0.35 m^3^  CH_4_/kg COD_consumed_), to the presence of high sulfate concentrations in the vinasse (5,336 mg/L).

The chloride concentration was measured in the residual media according to the addition of HCl to precipitate the rhamnolipid, in which a wide range of concentrations was verified. These chloride values imply sodium concentrations of 1.6–10.6 g/L in the neutralization previous to the anaerobic digestion stage. At high concentrations, sodium can affect the activity of microorganisms and interfere with their metabolism. Sodium concentrations are moderately (3500–5500 mg/L) to strongly (8000 mg/L) inhibitory at mesophilic temperatures. However, IC_50_ for sodium inhibition vary between 5.6 and 53 g/L due to the adaptation of microorganisms, antagonistic/synergistic effects with other cations, substrate type, and reactor configuration [40]. Again, the substitution of acid precipitation by innovative biosurfactant recovery methods is necessary.

The low pH value of the raw vinasse is due to the high concentration of volatile organic acids (13,080 mg/L as acetic acid) and no alkalinity. This characteristic is detrimental to anaerobic digestion because it indicates low buffering capacity of the medium and high probability of reduced pH and complete inhibition of the methanogenic activity. Total volatile acids (TVA)/alkalinity (ALK) values greater than 0.3 indicate the occurrence of disturbances in the anaerobic digestion [27]. After fermentation, except for two media (with TVA of 11.3 and 29.5 g/L), residual media showed TVA values of 56%, on average, smaller than the diluted raw vinasse, in addition to higher alkalinity values, which contribute to lower TVA/ALK ratios (average of 0.52) and are favorable to anaerobic digestion.

### 3.4. Anaerobic Biodegradability of Residual Fermentation Media

To assess whether biosurfactant production introduces some inhibitory effect on the anaerobic digestion of residual effluents from fermentation, four anaerobic biodegradability tests were carried out with six residual fermentation media generated in the production stage of the BS-V. In each assay, a control assay with raw vinasse was conducted for comparison.

**Figure 2:**
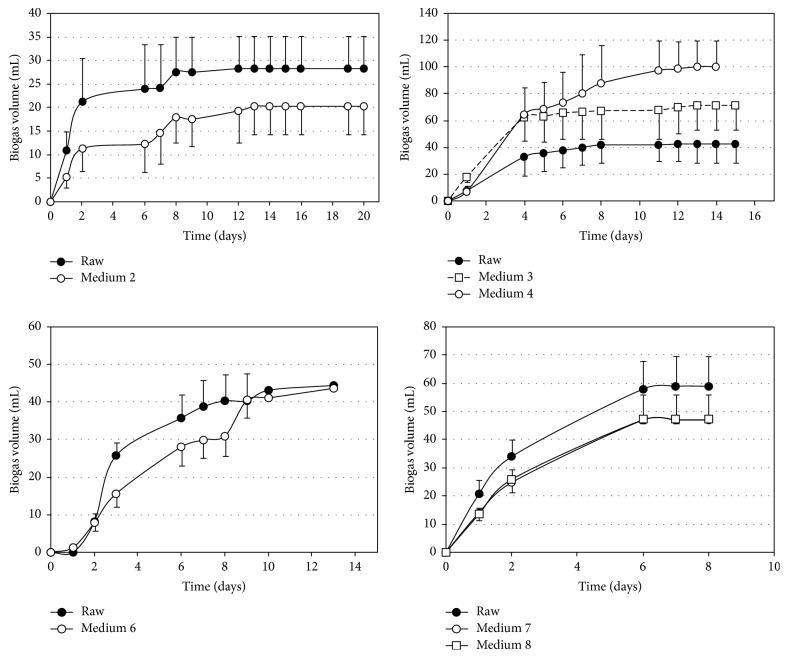
Biogas production evolution (30°C) in the anaerobic digestion of raw vinasse and residual media of submerged fermentation.

Figure 2 shows the biogas production results over time. The average biogas production using crude vinasse samples increased over the course of experiments, with minimum value of 28.3 ± 7.0 mL and maximum value of 59.0 ± 10.6 mL. This increased biogas production is due to the effect of higher concentrations of suspended solids in vinasse used in the first fermentations.

When vinasse supernatant was used, fermentations showed better results, as well as residual media in anaerobic digestion. The residual fermentation media behaved differently depending on composition variations. Medium 2 showed the lowest biogas production, with average replica value of 20.3 ± 6.0 mL, while medium 4 showed the highest biogas production, with 100.5 ± 19.4 mL. Media 6, 7, and 8 showed similar biogas production, with average values of 43.8 ± 4.3, 47.0 ± 1.0, and 47.2 ± 9.0 mL, respectively. Medium 2, despite its high biodegradability (BOD_5_/COD 0.5), had lower concentration of soluble COD compared to other media, indicating a smaller amount of substrate for biogas conversion, unlike medium 4, which had the highest concentration of soluble COD of all media studied.

Table 5 presents the results of four anaerobic biodegradability trials conducted, as well with crude vinasse (Control) in terms of pH, COD removal, biogas volume, and specific methane production. Medium 4, with higher biogas production, had specific methane production (SMP) less than the other residual media. Probably, the lower dilution of this residual medium, which led to initial COD value well above the desired value, allowed a higher salt concentration in the anaerobic digestion and methanogenesis inhibition.

Residual media 3 and 6, by contrast, showed higher SMP values because they had conditions favorable for anaerobic digestion, such as high concentration of soluble COD, low nitrogen concentration, and low salinity. Both media were derived from fermentation that required nitrogen supplementation (NH_4_OH or NaNO_3_) with C : N 60 mol/mol.

Medium 7, derived from fermentation that did not receive nitrogen supplementation, with C : N of 20 mol/mol and 168 h, which was a much shorter time than that adopted in media 3 and 6, showed good COD removal (63.2%) and slightly lower SMP (97.6 mL CH_4_/g COD_removed_). The reduced fermentation time and the lack of need for nutrient supplementation reduce costs and help enable the integrated process of biosurfactant production and anaerobic digestion of vinasse; therefore, medium 7 seems to be more suitable for biosurfactant production with medium containing added vinasse. A comparison of biogas production curves of control and medium 7 shows similar results for initial biogas production rate (17 mL/d for control and 13 mL/d for medium 7), stabilization time (7 d for control and 6 d for medium 7), and absence of lag phase in both groups (Figure 2).

Average COD removal and SMP values considering all control and residual media tests (except for medium 4) were used to better compare treatment with vinasse before and after fermentation for biosurfactant production. A comparison with literature data is difficult due to the different vinasse composition, operating conditions, and bioreactor type, with varying values. España-Gamboa et al. [41] achieved 69% COD removal and 0.263 m^3^  CH_4_/kg COD_added_ in the treatment of vinasse from ethanol production in modified UASB reactor fed with volumetric organic load of 17.05 kg COD/m^3^·day. Yeoh [42] achieved 65% COD removal and only 0.055 m^3^  CH_4_/kg COD_added_ treating cane-molasses alcohol vinasse in a thermophilic bioreactor with load of 14.49 kg COD/m^3^·day.

However, despite the values obtained for COD removal and SMP, the differences between the average COD removal for control (74.7 ± 7.4%) and residual media (65.4 ± 6.5%) in four anaerobic biodegradability tests are very close (9.3% difference) to standard deviations (7.4 and 6.5%), while the differences between the average SMP for control (99.7 ± 16.4 mL/g COD_removed_) and residual media (102.9 ± 9.0 mL/g COD_removed_) are lower (difference of 3.2 mL/g COD_removed_) than the standard deviations (16.4 and 9.0 mL/g COD_removed_). This comparison demonstrates that biosurfactant production does not interfere with wastewater treatment and methane production in the stage subsequent of anaerobic digestion, with similar COD removal and SMP values.

## 4. Conclusions

The production of rhamnolipid-type biosurfactant through submerged fermentation of* Pseudomonas aeruginosa* PA1 with vinasse-based medium is viable and produces fermented medium with lower rhamnolipid concentration but with surface tension and CMC similar to biosurfactant obtained by conventional medium. The residual medium derived from 168 h fermentation with vinasse diluted to 1 : 1 and supplemented only with glycerol to C : N of 21 mol/mol presented the best COD removal (63.2%) and SMP (97.6 mL CH_4_/g COD_removed_) in the anaerobic digestion. The biosurfactant production reduced the organic matter concentration of vinasse and did not inhibit the subsequent anaerobic digestion process.

## Figures and Tables

**Table 1:** C and N supplementation of vinasse-based fermentation media evaluated for biosurfactant production.

Medium	Carbon source	Nitrogen source	C/N (mol/mol)
1	Glycerol	NaNO_3_	60
2	Glycerol	NaNO_3_	60
3	Glycerol	NH_4_OH	60
4	Glycerol	NH_4_OH	60
5	Glycerol	NaNO_3_	60
6	Glycerol	NaNO_3_	60
7	Glycerol	—	21
8	Glycerol	NaNO_3_	15

**Table 2:** Physicochemical characteristics of raw vinasse and residual vinasse-based fermentation media.

Parameter	Unity	Raw Vinasse	Fermented media	
			Range	Average ± SD
pH	—	4.1	6.4–6.9	6.6 ± 0.2
VSS	mg/L	6594	334–639	466 ± 114
BOD_5_	g O_2_/L	57.6	8.5–9.8	9.2 ± 0.9
Total COD	g O_2_/L	77.0	14.6–45.8	30.3 ± 12.8
Soluble COD	g O_2_/L	60.4	13.8–39.4	24.8 ± 12.7
TOC	mg/L	27,362	—	
Total carbon	mg/L	28,600	—	
Total nitrogen	mg/L	1802	305–2077	998 ± 638
Total phosphorus	mg/L	15	nd–30	16 ± 33
Sulfate	g/L	5.8	1.6–11.6	5.3 ± 4.6
Chloride	g/L	3.5	2.5–16.4	6.7 ± 5.3
Volatile acids	g Acetic acid/L	13.1	1.8–29.5	8.7 ± 10.7
Alkalinity	g CaCO_3_/L	nd	1.3–57.0	16.9 ± 21.7

nd = not detected.

**Table 3:** Parameters of submerged fermentation of *Pseudomonas aeruginosa* PA1 in conventional medium and medium with vinasse and characteristics of biosurfactants produced under the same initial conditions.

Parameters/characteristics	BS-V	BS-C
Final time (h)	240	120
ΔRhamnolipids (g/L)	2.72 ± 0.12	3.22 ± 0.80
ΔBiomass (g/L)	1.90 ± 0.06	1.96 ± 0.04
ΔGlycerol (g/L)	19.34 ± 1.04	24.28 ± 1.53
*Y*_P/X_ (g/g)	1.43	1.64
*Y*_P/S_ (g/g)	0.14	0.13
*Q*_P_ (g/L·h)	0.011	0.027
*Q*_S(Gly)_ (g/L·h)	0.081	0.202
Surface tension (mN/m)	29.17 ± 0.06	28.64 ± 0.14
Critical Micelle Concentration (mg/L)	80.3	87.1

Δ, difference between final and initial concentrations of rhamnolipid, biomass, and glycerol; *Y*_P/X_, yield of rhamnolipid per unit of cell mass produced; *Y*_P/S_, yield of rhamnolipid per unit of substrate (glycerol) consumed; *Q*_P_, volumetric rhamnolipid production rate; *Q*_S(Gly)_, volumetric glycerol consumption rate.

**Table 4:** Cost of chemicals for production of 1-ton biosurfactant employing conventional fermentation medium (BS-C) or fermentation medium with vinasse (BS-V).

Chemicals	Unit Cost	Consumption	Total Cost US $	Consumption	Total Cost US $
		BS-V		BS-C	
NaOH 50% (m/v)	0.96 $/L	2.94 ml	0.00	0	0
NaNO_3_ 99.3% (m/m)	0.38 $/kg	1,760 kg	668.80	1,760	668.80
Glycerol 99.5% (m/v)	1.10 $/kg	7,153 kg	7,868.30	7,589 kg	8,347.90
KH_2_PO_4_ 98% (m/m)	1.20 $/kg	0	0.00	952 kg	1,142.40
K_2_HPO_4_ 98% (m/m)	1.20 $/kg	0	0.00	2,221 kg	2,265.20
MgSO_4_·7H_2_O 98% (m/m)	0.10 $/kg	0	0.00	63.5 kg	6.35
HCl 32% (m/v)	0.26 $/L	11.5 L	2.99	9.7 L	2.52

Total			8,540.09		12,433.17

BS-V: 2.72 g/L = 1 ton/368 m^3^ (vinasse supplemented with glycerol and NaNO_3_ – C/N 15); BS-C: 3.22 g/L = 1 ton/311 m^3^ (conventional medium – C/N 15).

**Table 5:** Summary of results of anaerobic biodegradability assays.

Trial	Sample	Final pH	Initial COD (mg/L)	Final COD (mg/L)	COD removal (%)	Biogas volume (mL)	Methane (%)	SMP average (mL CH_4_/g COD_removed_)
1	Raw	7.5 ± 0.1	3781	671 ± 22	82.2 ± 0.6	28.3 ± 7.0	na	na
	Medium 2	7.1 ± 0.1	3848	1002 ± 7	74.0 ± 0.2	20.3 ± 6.0	na	na
2	Raw	7.0 ± 0.1	3600 ± 35	792 ± 52	78.0 ± 1.4	42.8 ± 13.7	48.2 ± 9.7	81.20
	Medium 3	6.8 ± 0.1	5627 ± 106	1711 ± 70	69.6 ± 1.2	71.3 ± 17.9	55.9 ± 3.8	113.07
	Medium 4	6.5 ± 0.0	7597 ± 64	263 ± 31	96.5 ± 0.4	100.5 ± 19.4	48.7 ± 6.00	77.61
3	Raw	7.5 ± 0.1	3230	1134 ± 82	64.9 ± 2.5	44.5 ± 8.7	44.7 ± 2.4	105.38
	Medium 6	7.1 ± 0.1	3950	1692 ± 101	57.2 ± 2.6	43.8 ± 4.3	49.5 ± 1.3	107.56
4	Raw	7.3 ± 0.0	4917	1295 ± 46	73.7 ± 0.9	59.0 ± 10.6	62.1 ± 12.4	112.41
	Medium 7	7.2 ± 0.0	4157	1530 ± 79	63.2 ± 1.9	47.0 ± 1.0	49.1 ± 3.7	97.59
	Medium 8	7.3 ± 0.0	4513	1663 ± 122	63.1 ± 2.7	47.3 ± 9.0	50.8 ± 7.9	93.54

na: not analyzed.

## References

[1] Wilkie A. C., Riedesel K. J., Owens J. M. (2000). Stillage characterization and anaerobic treatment of ethanol stillage from conventional and cellulosic feedstocks. *Biomass & Bioenergy*.

[2] Cavalett O., Junqueira T. L., Dias M. O. S. (2012). Environmental and economic assessment of sugarcane first generation biorefineries in Brazil. *Clean Technologies and Environmental Policy*.

[3] UNICA [internet] (2016). *Total Ethanol Production Report 2015/2016*.

[4] España-Gamboa E., Mijangos-Cortes J., Barahona-Perez L., Dominguez-Maldonado J., Hernández-Zarate G., Alzate-Gaviria L. (2011). Vinasses: Characterization and treatments. *Waste Management & Research*.

[5] Jiménez A. M., Borja R., Martín A., Raposo F. (2006). Kinetic analysis of the anaerobic digestion of untreated vinasses and vinasses previously treated with Penicillium decumbens. *Journal of Environmental Management*.

[6] Pant D., Adholeya A. (2007). Biological approaches for treatment of distillery wastewater: A review. *Bioresource Technology*.

[7] Prado R. D. M., Caione G., Campos C. N. S. (2013). Filter cake and vinasse as fertilizers contributing to conservation agriculture. *Applied and Environmental Soil Science*.

[8] Ortegón G. P., Arboleda F. M., Candela L., Tamoh K., Valdes-Abellan J. (2016). Vinasse application to sugar cane fields. Effect on the unsaturated zone and groundwater at Valle del Cauca (Colombia). *Science of the Total Environment*.

[9] Parnaudeau V., Condom N., Oliver R., Cazevieille P., Recous S. (2008). Vinasse organic matter quality and mineralization potential, as influenced by raw material, fermentation and concentration processes. *Bioresource Technology*.

[10] Gunkel G., Kosmol J., Sobral M., Rohn H., Montenegro S., Aureliano J. (2007). Sugar cane industry as a source of water pollution - Case study on the situation in Ipojuca river, Pernambuco, Brazil. *Water, Air, & Soil Pollution*.

[11] Paredes D. D. S., Alves B. J. R., Dos Santos M. A. (2015). Nitrous Oxide and Methane Fluxes Following Ammonium Sulfate and Vinasse Application on Sugar Cane Soil. *Environmental Science & Technology*.

[12] Banat I. M., Makkar R. S., Cameotra S. S. (2000). Potential commercial applications of microbial surfactants. *Applied Microbiology and Biotechnology*.

[13] de Araujo L. V., Guimarães C. R., Marquita R. L. D. S. (2016). Rhamnolipid and surfactin: Anti-adhesion/antibiofilm and antimicrobial effects. *Food Control*.

[14] Kaskatepe B., Yildiz S. (2016). Rhamnolipid Biosurfactants Produced by Pseudomonas Species. *Brazilian Archives of Biology and Technology*.

[15] Henkel M., Müller M. M., Kügler J. H. (2012). Rhamnolipids as biosurfactants from renewable resources: Concepts for next-generation rhamnolipid production. *Process Biochemistry*.

[16] Lalov I. G., Krysteva M. A., Phelouzat J.-L. (2001). Improvement of biogas production from vinasse via covalently immobilized methanogens. *Bioresource Technology*.

[17] Moraes B. S., Zaiat M., Bonomi A. (2015). Anaerobic digestion of vinasse from sugarcane ethanol production in Brazil: challenges and perspectives. *Renewable & Sustainable Energy Reviews*.

[18] Salomon K. R., Lora E. E. S., Rocha M. H. (2011). Cost calculations for biogas from vinasse biodigestion and its energy utilization. *Sugar Industry*.

[19] Angenenta L. T., Karim K., Al-Dahhan M. H., Wrenn B. A., Domíguez-Espinosa R. (2004). Production of bioenergy and biochemicals from industrial and agricultural wastewater. *Trends in Biotechnology*.

[20] Pandey P., Shinde V. N., Deopurkar R. L., Kale S. P., Patil S. A., Pant D. (2016). Recent advances in the use of different substrates in microbial fuel cells toward wastewater treatment and simultaneous energy recovery. *Applied Energy*.

[21] Cherubini F. (2010). The biorefinery concept: Using biomass instead of oil for producing energy and chemicals. *Energy Conversion and Management*.

[22] Moraes B. S., Junqueira T. L., Pavanello L. G. (2014). Anaerobic digestion of vinasse from sugarcane biorefineries in Brazil from energy, environmental, and economic perspectives: Profit or expense?. *Applied Energy*.

[23] Greenberg A. E., Clesceri L. S., Eaton A. D. (2012). Standard Methods for the Examination of Water and Wastewater. *Water Pollution Control Federation*.

[24] Dilallo R., Albertson O. R. (1961). Volatile acids by direct titration. *Journal of the Water Pollution Control Federation*.

[25] Ripley L. E., Boyle W. C., Converse J. C. (1986). Improved alkalimetric monitoring for anaerobic digestion of high-strength wastes. *Journal of the Water Pollution Control Federation*.

[26] Santos A. S., Sampaio A. P. W., Vasquez G. S. (2002). Evaluation of different carbon and nitrogen sources in production of rhamnolipids by a strain of Pseudomonas aeruginosa. *Applied Biochemistry and Biotechnology*.

[27] Basandorj D. Anaerobic treatment of brewery wastewater using UASB (up flow anaerobic sludge blanket) reactors seeded with activated sludge.

[28] Santos A. S., Pereira N., Freire D. M. G. (2016). Strategies for improved rhamnolipid production by Pseudomonas aeruginosa PA1. *PeerJ*.

[29] Jamal A., Qureshi M. Z., Ali N., Ali M. I., Hameed A. (2014). Enhanced production of rhamnolipids by pseudomonas aeruginosa JQ927360 using response surface methodology. *Asian Journal of Chemistry*.

[30] Oliveira J. G. D., Garcia-Cruz C. H. (2013). Properties of a biosurfactant produced by Bacillus pumilus using vinasse and waste frying oil as alternative carbon sources. *Brazilian Archives of Biology and Technology*.

[31] Guerra-Santos L., Kappeli O., Fiechter A. (1984). Pseudomonas aeruginosa biosurfactant production in continuous culture with glucose as carbon source. *Applied and Environmental Microbiology*.

[32] Santa Anna L. M., Sebastian G. V., Pereira N. (2001). Production of biosurfactant from a new and promising strain of Pseudomonas aeruginosa PA1. *Applied Biochemistry and Biotechnology*.

[33] Elazzazy A. M., Abdelmoneim T. S., Almaghrabi O. A. (2015). Isolation and characterization of biosurfactant production under extreme environmental conditions by alkali-halo-thermophilic bacteria from Saudi Arabia. *Saudi Journal of Biological Sciences*.

[34] Lettinga G. (1995). Anaerobic digestion and wastewater treatment systems. *Antonie van Leeuwenhoek-Journal of Microbiology*.

[35] Reis R. S., Pacheco G. J., Pereira A. G., Freire D. M. G., Chamy R., Rosenkranz F. (2013). Biosurfactants: Production and Applications. *Biodegradation - Life of Science*.

[36] Desai J. D., Banat I. M. (1997). Microbial production of surfactants and their commercial potential. *Microbiology and Molecular Biology Reviews*.

[37] van Haandel A. C., van der Lubbe J. G. M. (2012). *Handbook of Biological Wastewater Treatment*.

[38] Grand View Researching [internet] (2015). *Biosurfactants Market Analysis by Product (Rhamnolipids, Sophorolipids, MES, APG, Sorbitan Esters, Sucrose Esters) and Segment Forecast to 2020*.

[39] Randhawa K. K. S., Rahman P. K. S. M. (2014). Rhamnolipid biosurfactants-past, present, and future scenario of global market. *Frontiers in Microbiology*.

[40] Chen Y., Cheng J. J., Creamer K. S. (2008). Inhibition of anaerobic digestion process: a review. *Bioresource Technology*.

[41] España-Gamboa E. I., Mijangos-Cortés J. O., Hernández-Zárate G., Maldonado J. A. D., Alzate-Gaviria L. M. (2012). Methane production by treating vinasses from hydrous ethanol using a modified UASB reactor. *Biotechnology for Biofuels*.

[42] Yeoh B. G. (1997). Two-phase anaerobic treatment of cane-molasses alcohol stillage. *Water Science and Technology*.

